# A prevalence-based approach to societal costs occurring in consequence of child abuse and neglect

**DOI:** 10.1186/1753-2000-6-35

**Published:** 2012-11-16

**Authors:** Susanne Habetha, Sabrina Bleich, Jörg Weidenhammer, Jörg M Fegert

**Affiliations:** 1 IGSF Institute for Health System Research GmbH, Schauenburgerstr, 116, 24118, Kiel, Germany; 2 Rehabilitation and Organization Division, Baden-Wuerttemberg Registered Hospital Association, Association of Hospitals, Rehabilitation- and Care Establishments, Birkenwaldstraße 151, Stuttgart, 70191, Germany; 3 Department of Child and Adolescent Psychiatry and Psychotherapy, University of Ulm, Steinhoevelstr. 5, Ulm, 89075, Germany

**Keywords:** Trauma follow-up costs, Trauma-related disorder, Cost of illness, Societal costs, Childhood traumatization, Child abuse, Child neglect, Child maltreatment

## Abstract

**Background:**

Traumatization in childhood can result in lifelong health impairment and may have a negative impact on other areas of life such as education, social contacts and employment as well. Despite the frequent occurrence of traumatization, which is reflected in a 14.5 percent prevalence rate of severe child abuse and neglect, the economic burden of the consequences is hardly known. The objective of this prevalence-based cost-of-illness study is to show how impairment of the individual is reflected in economic trauma follow-up costs borne by society as a whole in Germany and to compare the results with other countries’ costs.

**Methods:**

From a societal perspective trauma follow-up costs were estimated using a bottom-up approach. The literature-based prevalence rate includes emotional, physical and sexual abuse as well as physical and emotional neglect in Germany. Costs are derived from individual case scenarios of child endangerment presented in a German cost-benefit-analysis. A comparison with trauma follow-up costs in Australia, Canada and the USA is based on purchasing power parity.

**Results:**

The annual trauma follow-up costs total to a margin of EUR 11.1 billion for the lower bound and to EUR 29.8 billion for the upper bound. This equals EUR 134.84 and EUR 363.58, respectively, per capita for the German population. These results conform to the ones obtained from cost studies conducted in Australia (lower bound) and Canada (upper bound), whereas the result for the United States is much lower.

**Conclusion:**

Child abuse and neglect result in trauma follow-up costs of economically relevant magnitude for the German society. Although the result is well in line with other countries’ costs, the general lack of data should be fought in order to enable more detailed future studies. Creating a reliable cost data basis in the first place can pave the way for long-term cost savings.

## Background

### Childhood traumatization

Traumatization of children (the United Nations Convention on the Rights of the Child defines a "child" as "a human being below the age of 18 years") occurs in many ways. Due to their often very pronounced aftereffects, sexual, physical and emotional abuse in the home environment play a central role. For example, Maercker et al. [1] describe a Post-Traumatic Stress Disorder after sexualized violence in more than one third of the cases and Steil and Straube [2] in up to 80% of the cases. Close relationship with the offender, repetitions and combinations of various forms of abuse significantly contribute to this strong impact on the individual [3-6].

All in all, childhood traumatization is not a rare event. In two German studies on juveniles and young adults, 25.5% of the male and 17.7% of the female participants [7], or a total of 22.5% of the investigated juveniles [8] had already experienced at least one traumatic event. The most common types of traumatic events were violent attacks, accidents and witnessing trauma. Child neglect – mostly subdivided into physical and emotional neglect – also belongs to the most common types of traumatization [4,9,10].

A recent representative population survey in Germany [10] has shown that emotional abuse concerned 14.9%, physical abuse 12.0% and sexual abuse 12.5% of the respondents, where 14.5% were affected by “severe/extreme” abuse or neglect, respectively. In a somewhat older investigation by Wetzels [6], 38.8% of the participants had experienced physical violence by the parents more often than rarely, while 4.7% were severely affected. Contact sexual abuse before the age of 18 was indicated by 6.5% of the respondents (3.2% of men and 9.6% of women). A share of 8.9% of the participants had witnessed violence between the parents more than rarely.

A characteristic feature of childhood traumatization is that children are often affected by several types of traumatization. This has been shown for Germany [6,10] and in large population studies for Canada [11] and the USA [12,13]. The rate of children who were affected by at least two types of traumatization is presented with 40% in a German study [10] and with one quarter in a US-American study [13].

### Consequences of childhood traumatization

The consequences of childhood traumatization (hereinafter referred to as trauma-related disorders) are – apart from acute injuries and reactions – additionally mirrored in restricted social, emotional and physical development, which are associated with an increased risk of mental disorders, alcohol- and drug abuse as well as physical diseases in adulthood [14]. The “Final Report of the Independent Commissioner for Accounting for Child Sexual Abuse, Dr. Christine Bergmann” [15] published in May 2011 clearly demonstrates – especially by the integrated statements of the people concerned – how severely childhood traumatization can affect later life. With regard to the consequences of abuse, somatic complaints were reported starting at a rate of 50%, followed by relationship- and partnership problems, a row of symptoms typical of post-traumatic stress, performance impairment in connection with poorer school results, problems in professional education, occupational disability, etc., problems with self-esteem, self-hatred and self-disgust up to problems with corporeality and sexuality in even more than one fifth of the persons concerned.

Besides Post-Traumatic Stress Disorder (PTSD) – a trauma-related disorder by definition – increased risks after childhood traumatization have been shown and connections proven, respectively, in the following selected mental illnesses in retrospective and partly also prospective studies: depressive disorders [7,9,12,13,16-19], anxiety disorders [7,12,16,17,20], addictions [7,12,13,16-18,21,22], somatoform disorders [7,9,23-28], personality disorders [12,18,29-31] and conduct disorder [12,16,17,29]. The same applies to the following somatic diseases: overweight [3,13,19,32,33], diabetes mellitus [13,32,34,35], hypertension [36,37] and ischemic heart diseases [13,38-41]. The strength of the association between traumatization and trauma-related disorder reported in the aforementioned studies varies due to different approaches and methodologies.

Trauma-related disorders are thus by no means limited to the time of traumatization; they often accompany patients throughout their lives, with the time lag to the trauma being highly variable. While PTSD is defined over a close time connection of several weeks or months [42], trauma-related disorders such as depression, anxiety disorders, addiction or obesity can occur even in adulthood, i.e., years to decades after traumatization [3]. The far-reaching consequences of trauma-associated developmental disorders [43,44] and the multifaceted manifestation of trauma-related disorders, which affect various areas of life such as education, social contacts and working ability, are also mirrored in the high comorbidity rates that have been proven comprehensively for mental and somatic disorders in large studies [5,7,12,13,45-47].

However, childhood traumatization can by no means be regarded as the sole cause of the development of diseases or disorders [43]. Traumatization represents one of several variables in a biopsychosocial model, which measurably increases the risk of suffering from a certain disease or disorder. The extent of this risk increase varies depending on the type and severity of traumatization, individual conditions (e.g. gender) and the influence of external risk- and protective factors [2,23,48-50].

### Costs of childhood traumatization

Up to now, there are only few sources examining the cost side of childhood traumatization and the relevant social- and health policy questions. The total societal costs incurring due to childhood traumatization (hereinafter referred to as trauma follow-up costs) are unknown. A few studies from English-speaking countries have attempted to give at least approximate estimates of trauma follow-up costs. Authors of a cost-benefit analysis by the *National Center of Early Assistance* (*NZFH, Nationales Zentrum Frühe Hilfen*) [51] express concern that national studies on trauma follow-up costs are neither available nor feasible in Germany due to the non-availability of data.

What all studies known to the authors have in common is great uncertainty and incompleteness of the available data – starting from the prevalence of traumatization over the definition of cost areas to the calculation and allocation of costs. The variability of individual progressions between complete resilience and lifelong trauma-related disorders must be estimated as well, since they directly determine the long-term follow-up costs. By means of their conservative calculation methodology, studies on trauma follow-up costs explain in detail that the results of this puzzle are throughout underestimating [52-58]. In summary, underestimation is most notably explained by the insufficient availability of relevant data, which leaves certain cost areas partially or completely out of consideration, and by the uncertainty with regard to trauma prevalence, the dark figure of which (estimated number of unknown cases) can at best be taken into consideration but approximately.

One US-American study estimates societal costs related to reported cases of child abuse and neglect at USD 103.8 billion per year – without taking intangible costs into consideration [54]. For Australia, trauma follow-up costs have been calculated for the year 2007 in the amount of approximately AUD 4.0 billion on the basis of a population survey and in the amount of AUD 10.7 billion on the basis of prevalence information from literature [53]. In Canada, the annual amount is around CAD 15.7 billion – calculated as "a minimum cost to society" [55]. In the US state of Michigan, two consecutive cost-benefit studies refer to costs in the amount of USD 823 million for the year 1992 [56] and USD 1.8 billion for the year 2002 [57]. A cost-benefit study conducted in the state of New York quotes USD 9.0 million per year for "catastrophic maltreatment" [58].

Sources about federal costs in Germany are not known to the authors. The objectives of the present study are to estimate societal trauma-follow up costs (including direct and indirect costs) in Germany for the first time and to compare the results to costs in Australia, Canada and the USA.

## Methods

### Derivation of the total trauma follow-up costs

This prevalence-based cost-of-illness study is performed from the societal perspective. This perspective comprises not only individual costs, but most of all costs borne by society, caused by expenses in cost sectors such as health insurance, social service or losses in added value. The costs include those directly linked to traumatization as well as short- and long-term costs occurring due to aftereffects (indirect costs). The insufficiency of the available data did not allow for any estimation of opportunity costs. In order to estimate annual trauma follow-up costs in Germany, already published, aggregated data was used. The cost derivation follows a bottom-up approach based on the following formula:

Number of "A" units x costs per unit "A" = Total cost of "A" units.

In this context, unit "A" represents a case of (former) child abuse and/or neglect, respectively, where trauma-related disorders incur additional, economical costs for a lifetime.

Prevalence data for the determination of the number of "A" units were taken from the most recent survey on the prevalence of child abuse and neglect in Germany [10]. The survey provides up-to-date results on a good quality level, main features are summarized in Table 1.

**Table 1:** **Characteristics of the German prevalence study**[10]

	**Study characteristics**
Study type	Retrospective population survey
Objective	Prevalence of child maltreatment (physical, emotional, sexual) and neglect (physical, emotional) in Germany
Sample	Random sample
	Representative of the German population
	Sample size: 2,504
	Females: 53.2%, males 46.8%
	Age: 14–90 years, mean value: 50.6 years
Methods	Assessment of child maltreatment and neglect through the *Childhood Trauma Questionnaire* (German version)

The costs per unit "A" were obtained from the "Expertise on Cost-Benefit of Early Assistance" ("*Expertise Kosten und Nutzen Früher Hilfen*") by Meier-Gräwe and Wagenknecht [51]. To the knowledge of the authors, it is the first study of direct and indirect trauma follow-up costs in Germany that comprises a long lifespan up to the age of 67 years and which has been done in a very detailed and comprehensive way with respect to modeling costs. Study characteristics are shown in Table 2. Due to the large uncertainties resulting from the lack of reliable data, Meier-Gräwe and Wagenknecht have chosen a case-by-case approach for their cost-benefit-analysis. The result is four case scenarios for the representation of trauma follow-up costs, two "cheaper", moderate cases and two "expensive", pessimistic ones.

**Table 2:** **Characteristics of the German cost study**[51]

	**Study characteristics**
Study type	Cost-benefit-analysis
Objective	Assessment of cost and benefit of an Early Family Assistance Program
Methods	One prevention scenario versus four scenarios under the assumption of early child endangerment (child abuse and neglect fall within this definition)
	Case scenarios based on literature and expert knowledge
	Costs modeled on the basis of available data, supplemented by literature
Cost year	2008
Cost types	Healthcare services, social services, educational services and losses in productivity (due to low professional qualification, unemployment and occupational disability)
Direct Costs	Different types of educational family/parent support and foster care
Indirect Costs	Treatment of trauma-related disorders, educational services and productivity losses

In order to account for uncertainty, sensitivity analysis was performed by estimating a frame of trauma follow-up costs, based on the two different scenario types. Because of the great uncertainty of the information base and the lack of alternative resources, this cost-of-illness study follows a conservative approach. In order to abide by this principle and to create a coherent age range several adjustments had to be applied to prevalence and to cost data.

At first, the prevalence rate [10] was transferred to the German population utilizing population data from the German Federal Statistical Office [59]. From the given age groups the range of 15 to 64 years was the one that conformed best with the age ranges of the prevalence rate (≥ 14 years) and cost data (3 to 67 years). The exclusion of individuals aged 65 years and more is not expected to have any relevant influence on the prevalence rate, because only physical neglect was associated with a slightly higher risk in elderly persons (OR 1.03) [10].

Secondly, due to the individually different histories after traumatization, it cannot be assumed that all traumatized persons will suffer lifelong aftereffects [23,50], in particular not in an extent that would incur further costs in the dimension described later. Therefore, only the prevalence of "severe/extreme" cases as defined by the *CTQ*[10] was considered.

Yet even for the group of "severely/extremely" affected, it is not clear to what extent the consequences of trauma are reflected as measurable costs. Since one of the few available German studies [4] estimates the frequency of permanently impaired children among severely affected cases in child protection centers to be 21% (including cases of developmental retardation and learning disability), the authors have decided to use this 21% rate for derivation.

Furthermore, the case costs were adjusted for the age range of 15 to 64 years as defined by population data. As a consequence, the matters of expense in the years below and above that age range were deducted. This step was made on the assumption that costs are homogeneously distributed throughout the highest age group (51 to 67 years), whereas in the age group of 13 to 16 years the single matter of expense is considered in relation to the corresponding age.

Finally, the case costs were converted into annual costs by dividing them by the age range of 50 years (15 up to including 64 years). The case scenarios are presented on the 2008 cost level [51]. Consequently, total trauma follow-up costs are quoted in Euro for the year 2008. Since other years' cost figures are not included, no discounting was applied.

### Calculation of international comparative values

For a comparison of German costs with results from other countries, three prevalence-based cost studies from Australia [53], the USA [52] and Canada [55] were selected. These studies contain detailed descriptions of the calculation procedures and data resources so that the results can be better assessed. The study characteristics are presented in Table 3.

**Table 3:** Characteristics of the Cost Studies used for International Comparison

Annual Costs* (million)	**Population**	**Costing Methodology**	**Cost Types**
Australia [53]:AUD 3,947**	One-year prevalence in 0-17-year-olds: 3.7% (based on a population survey on physical and sexual abuse)	Short- and long-term costs of physical, emotional and psychological, sexual abuse, neglect and witness of (or knowledge of) family violence by a combination of top-down and bottom-up methods	Health, Additional educational assistance, Productivity losses of child abuse survivors and due to premature death, Crime, Government expenditures on care and protection, Deadweight losses
Canada [55]:CAD 15,706	Lifetime prevalence in 15-64-year-olds: 30% total, 14.6% severe (based on a population survey on physical and sexual abuse); Lifetime prevalence in 0-14-year-olds: 6.89% (based on the number of investigated cases of physical, emotional, sexual abuse or neglect)	Short- and long-term costs of physical, emotional, sexual abuse, neglect and witnessing violence by a combination of top-down and bottom-up methods	Judicial, Social Services, Education, Health, Employment, Personal
USA [52]:USD 7,300	Number of 0-17-year-old abuse victims (sexual, emotional and physical) in the year 1990: 794,000 (based on a national study of recognized child abuse and neglect); equal to 1,24% of that age group [own calculation]	Short- and long-term costs of physical, sexual and emotional abuse by a combination of top-down and bottom-up methods	Productivity, Medical Care/Ambulance, Mental Health Care, Police/Fire Services, Social/Victim Services, Property Loss/Damage

*Excluding intangible costs.

**From the three presented results the "best estimate" was chosen for comparison.

Comparison is made on the basis of purchasing power parity. While the German cost study [51] calculates prices of the year 2008, the international studies refer to the years 1993 [52], 1998 [55] and 2007 [53], respectively. Therefore, in a first step the foreign currencies were converted into Euro using the respective year’s purchasing power parity and were adjusted for inflation in a second step. These two steps were applied both to total trauma follow-up costs as presented in literature and to per capita costs, which were obtained by dividing the total costs by the respective country’s population in the respective cost year. The conversion and adjustment rates are shown in Table 4.

**Table 4:** Rates used for Purchasing Power Parity Conversion and Inflation Adjustment

	**Year**	PPP* Euro [60]**, own calculation]**	**Inflation**[61]
Australia	2007	1.719532906	0.98
Canada	1998	1.202424923	0.85
USA	1993	0.99786743	0.78
Germany	2008	1	1

*PPP: Purchasing Power Parity.

Additionally, the international costs were calculated as notional total costs for Germany by multiplying per capita costs of the respective country by the German population. These figures serve as a complementary way of illustrating the results, in order to point out the cost dimension of traumatization in relation to other societal expenses.

By means of these methods, differences between the individual countries with regard to purchasing power, population size and currency shall be balanced, so that results can be compared in the form of single figures on one level.

## Results

### Total trauma follow-up costs

The prevalence rate of at least one form of child abuse or neglect classified as "severe/extreme" is 14.5% [10]. This 14.5% share transferred to the German population aged between 15 and 64 years (54.1 million in the year 2008 [59]), the number of people concerned would be 7.8 million.

On the basis of the indications available in literature, only a 21% share of the 7.8 million individuals affected by “severe/extreme” child abuse or neglect has been included in the derivation of costs. This percentage equals 1.6 million (or 3.0% of the population aged 15 to 64 years), which represent the number of units "A".

The costs of the moderate scenarios average to EUR 432,951 (mean value of EUR 424,005 and EUR 441,896) for the age range of three to 67-year-olds; of the pessimistic scenarios, the average costs are EUR 1,159,294 (mean value of EUR 1,243,002 and EUR 1,075,585) for the age range of six to 67-year-olds [51]. By adjusting the costs for the age range of 15 to 64 years, average costs are reduced to a total of EUR 335,421 (mean value of EUR 326,745 and EUR 344,096) in the moderate scenario and to EUR 904,375 (mean value of EUR 870,579 and EUR 938,169) in the pessimistic scenario. The resulting average annual costs, related to a period of 50 years, amount to EUR 6,708 per unit "A" in the moderate scenario and to EUR 18,087 in the pessimistic scenario.

Substituting the variables of the above described formula by figures of the cost margin’s lower bound (moderate scenario):

(1) 1,648,389x6,708Euro=11,057,396,330Euro,

the resulting total annual costs amount to EUR 11.1 billion, which incur as follow-up costs of child abuse and neglect, respectively, for German society. In other words, the annual per capita trauma follow-up costs would amount to EUR 134.84 (German population 2008: 82,002,400 [59]).

Applying the costs of the pessimistic scenario to the formula:

(2) 1,648,389x18,087Euro=29,814,419,711Euro,

the upper bound of the annual trauma follow-up cost frame is EUR 29.8 billion in total or EUR 363.58 per capita.

### International comparative values

The international, comparative values of per capita trauma follow-up costs (without intangible costs) are EUR 106.20 according to the Australian, EUR 22.14 according to the US-American, and EUR 368.16 according to the Canadian calculation each year (cf. Table 5). As notional total annual costs for the German society, these values would amount to EUR 8.7 billion (Australian calculation), EUR 1.8 billion (US-American calculation), and EUR 30.2 billion (Canadian calculation), respectively.

**Table 5:** Conversion of International Cost Values into Euro in the Year 2008

	**Original total annual costs in national currency**	**Total annual costs in Euro, different cost years**	**Total annual costs in Euro, 2008**	Per capita costs* in Euro, different cost years	Per capita costs* in Euro, 2008
Australia, without intangible costs [53]	3,947,000,000	2,295,390,793	2,249,482,977	108.37	106.20
Australia, including intangible costs [53]	10,691,000,000	6,217,386,108	6,093,038,386	293.54	287.67
USA, without intangible costs [52]	7,300,000,000	7,315,601,034	5,706,168,807	28.38	22.14
USA, including intangible costs [52]	56,000,000,000	56,119,679,168	43,773,349,751	217.70	169.81
Canada, without intangible costs [55]	15,705,910,047	13,061,863,365	11,102,583,860	433.13	368.16

*Australian population in the year 2007: 21,180,632 [62], Canadian population in the year 1998: 30,157,082 [63], US-American population in the year 1993: 257,783,000 [64]. Calculation was made – to the extent possible – prior to rounding of values.

The German lower bound in the amount of EUR 11.1 billion per year is close to the Australian result, while the Canadian study has returned costs very close to the German upper bound. The US-American study is somewhat out of scope with about one sixth of the German lower bound costs.

The Australian and US-American studies additionally quote intangible costs, whereby results are increased to EUR 287.67 and EUR 169.81, respectively, per capita (EUR 23.6 billion and EUR 13.9 billion total costs).

## Discussion

### Total trauma follow-up costs

The objective of the present study was to estimate trauma follow-up costs for Germany. A margin of total annual trauma follow-up costs was calculated in the amount of EUR 11.1 billion for the lower bound and EUR 29.8 billion for the upper bound, respectively. The correspondence of the Australian result with Germany’s lower bound should be considered with utmost caution, since both cost studies are based on completely different methods and also include different cost areas. In contrast to the Australian study [53], the German cost calculation [51] does not take crime and deadweight losses into consideration, whereas the areas health, education, productivity and social services have been considered equally.

In both studies, prevalence is based on a population survey with similar results for the lifetime prevalence of physical and sexual abuse (17.8% in Australia [53] and approximately 15.9% in Germany [10, own calculation]). However, the Australian study uses only the one-year prevalence of 0 to 17- year-olds and does not include emotional abuse or neglect. Thus, the number of people concerned is much lower in the Australian study, whereas the total costs per person must lie close to those of Germany’s upper bound: the upper bound costs (EUR 18,087) multiplied by Australia’s prevalence (3.7% of Germany’s population aged 0–15 years: 412.147, age range as presented by the German Federal Statistical Office [59]) would yield – with EUR 7.5 billion – a result quite close to the Australian one.

The Canadian result [55] is very close to the German upper bound, but relies on higher prevalence rates (cf. Table 3), which have been used for cost calculation in a sophisticated way. Canadian costs comprise expenses related to the legal system, social services, education, health, employment and personal costs, with the employment sector being the most expensive one, accounting for 72% of the total costs (CAD 11.3 billion of a total of CAD 15.7 billion). Other than in the Australian and US-American studies, Canada has based their cost calculation in the employment sector on a large population survey, which combined information on income with physical and sexual abuse in the respondents' history (*Ontario Health Survey Mental Health Supplement (OHSUP)*).

With a loss of productivity of over 70% in the moderate case scenarios and over 50% in the pessimistic ones, the German cost-benefit-analysis [51] ranks close to the Canadian result. Since costs are oriented towards individual life courses in both countries – in Canada on the basis of a population survey and in Germany on the basis of individual case scenarios – this result could in fact point in the right direction, namely to regarding productivity losses as the main cost driver of societal trauma follow-up costs. The other two studies [52,53] rely on less specific, aggregated data. In Australia [53], losses of productivity rank far behind the other areas, while an approximate proportion of almost 30% can be derivated for the United States [52].

The total costs in the United States [52] are considerably lower than those in other countries, even though the cost sectors taken into consideration largely correspond. However, on the one hand, child neglect is not included for methodological reasons, and on the other hand, the number of child abuse victims is not determined on the basis of a population survey, but official information sources are used [65]. Despite the attempt to calculate institutionally unknown cases, the dark figure remains largely unconsidered. There is naturally no precise information regarding the magnitude of this dark figure. Wetzels [6] indicates an optimistic estimate at the ratio of one to ten. This estimate projected on the US-American study would yield a result of EUR 18 billion instead of EUR 1.8 billion and thus above the Australian costs and within the German cost frame.

The two results from Australia [53] and the United States [52], which contain intangible costs, cannot be compared with the German result (without intangible costs). In the Australian study, intangible costs make up for 1.7 times, in the US-American study even 6.7 times of the tangible costs. In spite of this large difference it can be stated on the transnational level that intangible costs as a measure for personally experienced burden considerably exceed the actual expenses in the form of tangible costs in any case.

Generally, the comparison of the four aforementioned results of trauma follow-up costs is limited due to the time lag of altogether fifteen years between the individual studies, which have certainly influenced prices, services and their utilization. Additionally, national service organization and funding structures, e.g. of the health care systems, are fundamentally different [66]. These variations presumably influence the availability and the assessment of costs and their assignment to various sectors, so that differences in costs are to be expected *a priori* due to structural conditions.

The calculated amount of trauma follow-up costs clearly has economic relevance, constituting 0.44% (lower bound) and 1.20% (upper bound) of Germany’s 2008 Gross Domestic Product (EUR 2,489.4 billion) [67]. The figures have an additional relevance due to the fact that with early and effective intervention or prevention, they reveal a considerable saving potential [9,51,56-58].

Basically, trauma follow-up costs were determined by following a conservative approach. This is reflected in several details, for example in the restriction to a 21% share of only severely affected cases [4,10]. Results of risk- and resilience research lie around this value for the share of traumatized individuals with long-term consequences caused by trauma-related disorders [50,68].

Moreover, total costs have only been taken into account for the age group from 15 up to including 64 years. Consequently, direct costs are only considered to a small extent and indirect costs of older ages remain completely excluded. With existing trauma-related disorders, it can be assumed that the age-related, increasing instability of life situation leads to further health problems, which again incur additional costs in higher age. In general, trauma-related disorders do not tend to decrease in higher age [1,69], but elderly people are often severely impaired due to e.g., insufficient specialized care [15].

Last but not least it should be noted that types of traumatization other than sexual, physical and emotional abuse and neglect are left unconsidered in the present study, so that no statements can be made on their prevalence or on follow-up costs. However, it appears reasonable to limit the derivation of trauma follow-up costs to child abuse and neglect, since other current data are not available and international cost studies [52-58] refer to these types of traumatization exclusively or predominantly, so that results can be better compared with each other.

When trying to estimate whether the true costs may tend towards the lower or the upper bound one has to keep in mind that the cost scenarios are based on early childhood traumatization, whereas the prevalence data include the entire childhood and adolescence as defined by the *CTQ*. Since trauma-related disorders tend to be more severe the earlier traumatization was experienced [2,70], this discrepancy leads in the direction of the lower bound.

The international comparison supports both the lower and the upper bound of the cost margin – depending on the respective study. Due to methodological differences the results have to be interpreted rather as crude reference points, though. Despite all limitations, the comparison shows that the cost margin calculated for Germany is well associated with other countries’ results.

### Limitations

Limitations associated with the use of already existing data are particularly given by the fact that these data have been collected with different objectives and are not well-matched. The question arises, in particular, to what extent the cost scenarios – determined under the assumption of child endangerment [51] – can be projected on the number of traumatized individuals identified in epidemiologic studies [4,10]. While various age limits of the investigated populations can be approximated, it cannot be stated with certainty whether the cost scenarios described by Meier-Gräwe and Wagenknecht [51] are based on the same kind of traumatization as the determination of prevalence by Häuser et al. [10].

The prevalence of traumatization has been determined by Häuser et al. [10] retrospectively, which may represent yet another source of error – due to blurred or imprecise memories. However, several studies of this kind illustrate the fact very well that the number of errors is to be estimated rather low and of conservative type, in other words, that the results tend to underestimate reality [3,5,11,20,45,71,72].

Another significant uncertainty lies in the cost data themselves. The authors of the cost study explain in detail that due to the lack of data, several parts had to be completed by expert knowledge and international literature [51]. The complete case scenarios are thus but a construct, which has been developed as close to reality as possible with the help of various information sources.

The problem of low availability and unsatisfactory quality of the data with regard to the estimation of trauma follow-up costs does not only exist in Germany but it is criticized in all cost studies [52-58]. Consequently, results are consistently presented as fragmentary and underestimating. Since it can therefore be assumed that all cost studies deviate from reality in the same direction – with the extent of deviation being unclear – a comparison is possible and reasonable. Nevertheless, it can only be valued as a comparison of cost dimensions, not of amounts calculated precisely to the cent, solely due to the different methodologies. In view of the generally weak data, it should be noted that by using more precise procedures, only the illusion of higher precision could be created. This is not the intention of the authors.

### Perspective

Realizing numerous questions and imponderabilities in the assessment of results, creating a reliable data basis must be of highest priority in Germany, in order to answer the question how expensive it is not to provide sufficient and timely assistance to traumatized children and juveniles. The gathering of reliable cost data seems to be a highly challenging task in the light of an extremely fragmentary information basis. Serious efforts should therefore be undertaken to collect reliable data, in the first place. Only on the basis of results that are accepted by all sides due to their validity can steps be ground in order to sustainably improve the status quo of prevention and post-traumatic care.

Starting points for the improvement of care and thus assumingly also for long-term cost savings are indicated in numerous literature sources, which, for example demand a stronger interconnectedness of the respective institutions [15,73-77] or a more specific qualification in the medical community [15,78-81]. Fiscally responsible decision-making, though, should rely on the economic evaluation of any intervention or prevention program [82].

## Conclusions

Total costs of EUR 11.1 billion or EUR 29.8 billion, respectively, for the consequences of childhood traumatization by various types of severe child abuse as well as neglect are undoubtedly relevant for German economy. Considering the paucity of data, especially of cost data, the result cannot be seen without restrictions. Therefore serious efforts should be undertaken to generate reliable data, in the first place.

Besides the question of personal suffering, political decision-makers should pay much more attention to the economic perspective of childhood traumatization and its comprehensive dimension of long-term consequences. By improving trauma-related care and prevention, the societal economic burden might be reduced.

## Abbreviations

CTQ: Childhood Trauma Questionnaire; NZFH: National Center of Early Assistance (*Nationales Zentrum Frühe Hilfen*); PTSD: Post Traumatic Stress Disorder.

## Competing interests

The authors declare that they have no competing interests.

## Authors’ contributions

JMF and JW conceived the idea of the study, reviewed the manuscript and gave final approval of the version to be published. JMF obtained funding for the study. JW advised SH on the study design. SH conceived the calculation model and drafted the manuscript. SB gave methodological support and coordinated external and internal affairs. All authors have read and approved the final manuscript.

## Authors’ information

SH has worked as a medical doctor before additionally graduating in Public Health. She specialized in DRG-based hospital reimbursement while working in the German DRG-Institute (*Institut für das Entgeltsystem im Krankenhaus GmbH*). Currently, SH is working on different health economics-related projects.

SB holds a Master of Science and a PhD in Economics. During the progress of this paper she has worked as JMF’s Executive Assistant at the Hospital for Child and Adolescent Psychiatry and Psychotherapy of the Ulm University Hospital. Currently she is working at the *Baden-Württembergische Krankenhausgesellschaft*, a regional association of hospitals.

JW is neurologist and psychiatrist, specialized in psychotherapy and psychoanalysis. Over a period of many years he has gained comprehensive and multifaceted experiences in Health Management, e.g. as Medical Director or Health Management Consultant, most recently as Managing Director at *Asklepios Medical School GmbH*.

JMF is child and adolescent psychiatrist and psychotherapist. He is Medical Director and founder of the Hospital for Child and Adolescent Psychiatry and Psychotherapy at the Ulm University Hospital. He is member of the Scientific Board for Family Affairs at the *Bundesministerium für Familie, Senioren, Frauen und Jugend* (*BMFSFJ*), and since 2010 he is deputy chairman of this board.
